# Predictors of autosomal dominant polycystic kidney disease progression: a Brazilian single-center cohort

**DOI:** 10.1590/2175-8239-JBN-2023-0040en

**Published:** 2024-06-24

**Authors:** Igor Hitoshi Nishimoto, Andrey Gonçalves Santos, Júlia Mandelbaun Bianchini, Luiz Gustavo Brenneisen Santos, Maria Carolina Rodrigues Martini, Vanessa dos Santos Silva, Luis Cuadrado Martin

**Affiliations:** 1 Universidade Estadual Paulista "Júlio de Mesquita Filho", Escola de Medicina, Botucatu, SP, Brazil.; 2 Universidade Estadual Paulista "Júlio de Mesquita Filho", Escola de Medicina, Departamento de Medicina Interna, Botucatu, SP, Brazil.

**Keywords:** Polycystic Kidney, Autosomal Dominant, Renal Insufficiency, Rate, Mortality, Risk Factors

## Abstract

**Introduction:**

Identifying risk factors for autosomal dominant polycystic kidney disease (ADPKD) progression is important. However, studies that have evaluated this subject using a Brazilian sample is sparce. Therefore, the aim of this study was to identify risk factors for renal outcomes and death in a Brazilian cohort of ADPKD patients.

**Methods:**

Patients had the first medical appointment between January 2002 and December 2014, and were followed up until December 2019. Associations between clinical and laboratory variables with the primary outcome (sustained decrease of at least 57% in the eGFR from baseline, need for dialysis or renal transplantation) and the secondary outcome (death from any cause) were analyzed using a multiple Cox regression model. Among 80 ADPKD patients, those under 18 years, with glomerular filtration rate <30 mL/min/1.73 m^2^, and/or those with missing data were excluded. There were 70 patients followed.

**Results:**

The factors independently associated with the renal outcomes were total kidney length – adjusted Hazard Ratio (HR) with a 95% confidence interval (95% CI): 1.137 (1.057–1.224), glomerular filtration rate – HR (95% CI): 0.970 (0.949–0.992), and serum uric acid level – HR (95% CI): 1.643 (1.118–2.415). Diabetes mellitus - HR (95% CI): 8.115 (1.985–33.180) and glomerular filtration rate - HR (95% CI): 0.957 (0.919–0.997) were associated with the secondary outcome.

**Conclusions:**

These findings corroborate the hypothesis that total kidney length, glomerular filtration rate and serum uric acid level may be important prognostic predictors of ADPKD in a Brazilian cohort, which could help to select patients who require closer follow up.

## Introduction

Autosomal dominant polycystic kidney disease (ADPKD), the most common monogenic cause of end-stage kidney disease (ESKD), is characterized by inexorable development of kidney cysts, hypertension and destruction of the kidney parenchyma^1^. This disease is characterized by the formation of multiple cysts in the kidneys, whose growth leads to compression and ischemia of adjacent nephrons and an inflammatory process that results in fibrosis and progressive impairment of renal function.

The main causes of death in ADPKD patients are cardiovascular diseases^2^. High blood pressure is present in more than half of the patients before the decline in the glomerular filtration rate^3^ and is the main determinant of this outcome. The poor prognosis of ADPKD patients is related to larger size of the kidneys, male sex, poorly treated hypertension, and the PKD1 gene^4,5,6^. Black patients and those with hematuria before the age of 30, onset of hypertension before the age of 35, proteinuria and hyperlipidemia are also more likely to have a worse outcome^4,7^.

Furthermore, low levels of high-density lipoprotein (HDL) and high levels of cholesterol and low-density lipoprotein (LDL) have been identified as risk factors for the ADPKD progression^8,9,10,11^.

In ADPKD patients, glomerular filtration rate decreases over 10 to 20 years from the diagnosis, and about 60% progress to ESKD until the seventh decade of life^8^. The treatment of ADPKD is targeted mainly at symptoms and complications.

Given these points, it is extremely important to identify predictors of ADPKD progression, in order to follow patients at higher risk closely, while also mitigating the worsening of the disease and its complications. However, studies that have evaluated this subject among a Brazilian cohort have not yet been identified.

Thus, this study aims to identify risk factors looking for associations between clinical and laboratory variables with the renal outcomes and death in ADPKD patients followed among a Brazilian single-center cohort.

## Methods

A longitudinal study was carried out among a cohort of ADPKD patients, and this study was approved by the local ethics committee under number: 3,383,261. The medical records of all patients who had their first medical appointment at the Nephrology Service of the Medical School at Botucatu Clinical Hospital from January 2002 to December 2014 were consulted to find ADPKD patients. This was done through an active search for all imaging exams in the medical records. Total abdomen ultrasound (US), renal US and abdominal computed tomography (CT) were evaluated. These exams were carried out according to hospital routine without any specific standardization, since this study is a real-life work.

The diagnosis of ADPKD^12,13^ was considered:

- For individuals belonging to families affected by ADPKD: presence of three or more cysts, unilateral or bilateral, in patients between 15 and 39 years old; two or more cysts in each kidney in patients 40 to 59 years old, and four or more cysts in each kidney for patients over 60 years;
- In individuals with suspected ADPKD, but without a positive family history: presence of 20 or more cysts in each kidney, particularly if the kidneys are enlarged or extra-renal cysts, and in the absence of obvious features of other cystic diseases^14^.

We included in the study people with ADPKD according to the criteria above, and over the age of 18. Patients with estimated glomerular filtration rate (eGFR) <30 mL/min/1.73 m^2^, using the CKD-EPI equation, at the beginning of the follow-up and patients with incomplete data were excluded.

The patients were followed until December 2019. The primary outcome was sustained decrease of at least 57% in the eGFR from baseline (this decrease is equivalent to double the creatinine, which is a classical renal outcome)^15^, need for dialysis or renal transplantation, and the secondary outcome was death due to any cause. The independent variables were age, sex, race, the sum of the largest renal axis (total kidney length), smoking, weight, height, body mass index, presence of diabetes mellitus (DM), presence of coronary artery disease, presence of cerebrovascular disease, presence of peripheral artery disease and presence of atherosclerotic disease (coronary artery disease, cerebrovascular disease, or peripheral artery disease), all these variables at baseline. Systolic and diastolic blood pressure were considered the average of all available records. The following laboratorial data were evaluated at baseline: serum creatinine, estimated glomerular filtration rate (eGFR), serum potassium, calcium, phosphorus, sodium, total cholesterol, HDL, LDL, triglycerides, parathyroid hormone, C-reactive protein, and serum uric acid level. Hemoglobin, white blood cells, platelets, urinary volume, proteinuria, urinary density, presence of macroscopic hematuria and urinary 24-hour sodium were also evaluated.

Categorical variables were analyzed according to the chi-square test; continuous variables using the Students-t test if there was a normal distribution and the Mann-Whitney test when patients did not have a normal distribution. The results were listed in tables using values of mean and standard deviation or absolute and relative frequency. The variables that were associated with the outcomes at the level of p < 0.10 were included in the multiple Cox regression model. Collinearities were tested and, when present, the variable with the greatest clinical significance was chosen. Subsequently, automatic variable selection (backward stepwise) was used. An analysis of the ROC curve (Receiver Operating Characteristic Curve) was also used to evaluate the discriminatory power of the total kidney length in relation to the renal outcome. The Youden index (greater sum of specificity and sensitivity) was used to verify the best cut-off point, and positive and negative likelihood ratios were also calculated. The results were discussed at the level of p < 0.05.

## Results

**Figure 1.**
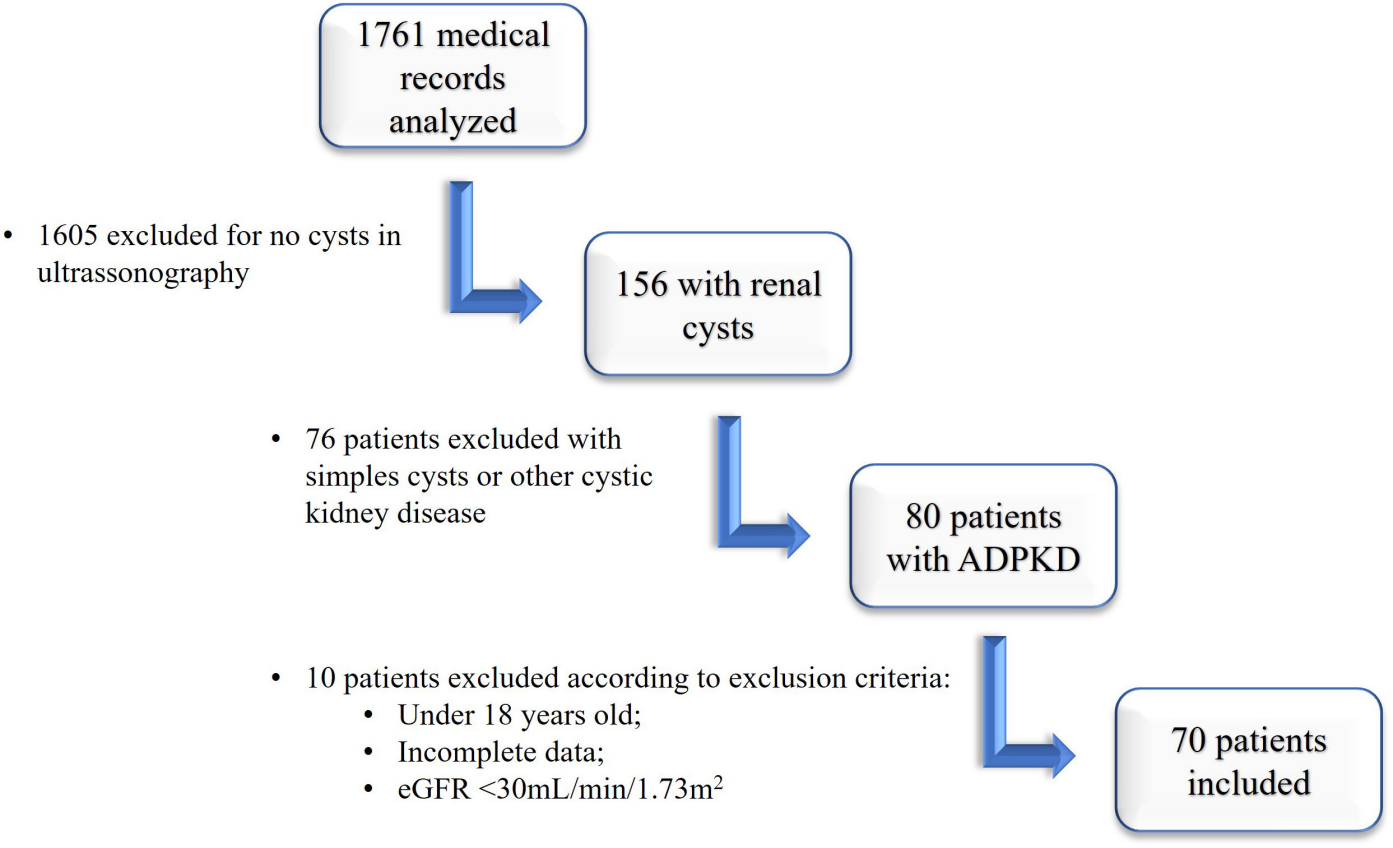
Flowchart of patient’s inclusion.

A total of 1761 medical records were consulted to find ADPKD patients. After reviewing all medical records, there were 156 patients with renal cysts. From the exclusion of patients with simple cysts and other cystic kidney diseases other than ADPKD, the number of ADPKD patients obtained was 80. According to the exclusion criteria, we excluded six patients under 18 years and four with eGFR <30 mL/min/1.73 m^2^ at the beginning of the follow-up (Figure 1).

The cohort study was composed of 70 patients, with a mean age of 46 ± 16.1 years, 37 men (53%), and 6 non-white (9%). There were 65 patients submitted to ultrasonography and 5 patients submitted to CT scans. Most were active or inactive smokers (57%), 19% were diabetic, and 21% had some atherosclerotic disease. The follow-up period range was between 1.2 and 198 months, with a mean of 109 ± 55 months and a median of 110 (interquartile range: 71–158) months.

The renal outcome was observed in 23 patients. Total kidney length was statistically different between progressors and non-progressors (Table 1). Among the laboratory variables, serum creatinine, eGFR serum creatinine, HDL, serum uric acid level and urinary density were associated with the primary (renal) outcome (Table 2).

**Table 1.** Clinical data of patients with ADPKD in relation to renal outcomes (double-increased creatinine or entering dialysis) in a Brazilian cohort

	Renal outcome (n = 23)	Without renal outcome (n = 47)	p
Age* (Years)	47 ± 11.6	45 ± 18.1	0.532
Non-white people (%)	2 (9%)	4 (9%)	0.980
Men (%)	2 (9%)	4 (9%)	0.980
Smoking^#^ (%)	16 (70%)	24 (51%)	0.234
*Diabetes mellitus*	7 (30%)	6 (13%)	0.098
Weight (Kg)	76 ± 15.6	74 ± 14.5	0.691
Height (cm)	169 ± 11.2	167 ± 9.3	0.477
BMI (Kg/m²)	26.93 ± 4.16	25.08 ± 6.37	0.325
Presence of CAD	3 (13%)	5 (11%)	0.766
Presence of CVD	2 (9%)	4 (9%)	0.467
Presence of PAD	2 (9%)	4 (9%)	0.979
Atherosclerotic disease	5 (22%)	10 (21%)	0.964
SBP (mmHg)	137 ± 12.4	133 ± 11.5	0.180
DBP (mmHg)	84 ± 7.9	83 ± 8.1	0.148
Left Kidney (cm)	16.3 ± 3.61	14.0 ± 2.46	0.003
Right Kidney (cm)	16.3 ± 3.39	13.9 ± 2.79	0.003
Total kidney length (cm)	32.6 ± 6.62	27.8 ± 4.76	0.001

Abbreviations – BMI: body mass index, CAD: coronary artery disease, CVD: cerebrovascular disease, PAD: peripheral arterial disease, SBP: systolic blood pressure, DAP: diastolic blood pressure. Notes – *At the beginning of the follow-up, ^#^active or previous.

**Table 2.** Laboratory data on patients with ADPKD regarding renal outcomes (double-increased creatinine or entering dialysis) in a Brazilian cohort

	Renal outcome (n = 23)	Without renal outcome (n = 47)	p
Creatinine (mg/dL)	1.4 ± 0.43	1.0 ± 0.27	<0.001
CKD-EPI (ml/min/1.73m²)	61.1 ± 26.03	83.3 ± 25.77	<0.001
Potassium (mEq/L)	4.5 ± 0.56	4.4 ± 0.57	0.311
Calcium (mg/dL)	9.2 ± 0.75	9.5 ± 0.69	0.082
Phosphorus (mg/dL)	3.7 ± 0.57	3.6 ± 0.66	0.602
Sodium (mmol/L)	141.4 ± 1.75	141.2 ± 2.75	0.692
PTH (pg/mL)	88.3 ± 46.68	67.2 ± 54.11	0.141
Hemoglobin (g/dL)	13.3 ± 1.90	13.6 ± 1.58	0.602
Platelets (10³/mm³)	266 ± 116.3	237 ± 72.2	0.218
White blood cells (10^3^/mm^3^)	8.9 ± 5.50	7.7 ± 2.24	0.234
CRP (mg/dL)	1.1± 1.75	1.1 ± 1.12	0.955
Total cholesterol (mg/dL)	179.2 ± 36.85	176.2 ± 33.82	0.737
Triglycerides (mg/dL)	168.6 ± 61.58	141.0 ± 79.35	0.147
HDL (mg/dL)	39.4 ± 9.84	46.8 ± 10.47	0.006
Calculated LDL (mg/dL)	106.1 ± 32.72	101.3 ± 26.25	0.509
Proteinuria (g/24h)	0.04 ± 0.065	0.06 ± 0.173	0.686
Uric Acid (mg/mL)	6.7 ± 1.06	5.8 ± 1.40	0.008
Urinary volume (mL)	1929 ± 558.9	1742 ± 725.5	0.324
Urinary density (g/dL)	1011.2 ± 1.77	1013.9 ± 4.09	0.004
RBC/HPF	6.0 ± 10.94	5.0 ± 11.78	0.729
Urinary Sodium (mEq/24h)	147.1 ± 69.55	197.5 ± 87.55	0.303

Abbreviations – PTH: parathyroid hormone, CPR: C-reactive protein, HDL: high density protein; LDL: low density protein, WBC: white blood cells, RBC/HPF: red blood cells per high power field.

The variables above were selected for multiple Cox regression models (except serum creatinine, as it has a strong collinearity with glomerular filtration rate). Presence of DM was also selected to compose the multiple analysis. Using the backward stepwise selection, the final model was obtained in which there is an association between renal outcome and total kidney length, eGFR and serum uric acid level (Table 3). In the final adjusted model, each centimeter in total kidney length was associated with a renal outcome Hazard Ratio (HR) of 1.137, with a 95% Confidence Interval (95% CI) of 1.057–1.224, for each unit (mL/min/1.73 m^2^) of more glomerular filtration rate, HR (95% CI) of 0.970 (0.949–0.992) was obtained, and each unit (mg/mL) of serum uric acid level was associated with HR (95% CI) of 1.643 (1.118–2.415).

**Table 3.** Multiple Cox analysis with the renal outcome as an independent variable in a Brazilian cohort

		HR	95% CI		p
			Inferior	Superior	
Step 1	Total Kidney length (cm)	1.123	1.043	1.209	0.002
	CKD-EPI (mL/min/1.73 m^2^)	0.977	0.955	0.999	0.045
	Uric Acid (mg/mL)	1.555	0.996	2.426	0.052
	*Diabetes mellitus*	1.175	0.448	3.084	0.743
	HDL (mg/dL)	0.966	0.906	1.031	0.298
	Urinary density (g/dL)	0.889	0.730	1.083	0.244
Step 2	Total Kidney length (cm)	1.122	1.043	1.207	0.002
	CKD-EPI (mL/min/1.73 m^2^)	0.977	0.955	1.000	0.048
	Uric Acid (mg/mL)	1.550	0.996	2.413	0.052
	HDL (mg/dL)	0.963	0.906	1.024	0.234
	Urinary density (g/dL)	0.889	0.730	1.083	0.244
Step 3	Total Kidney length (cm)	1.123	1.043	1.210	0.002
	CKD-EPI (mL/min/1.73 m^2^)	0.972	0.952	0.993	0.009
	Uric Acid (mg/mL)	1.443	0.947	2.198	0.088
	HDL (mg/dL)	0.963	0.909	1.020	0.195
Step 4	Total Kidney length (cm)	1.137	1.057	1.224	0.001
	CKD-EPI (mL/min/1.73 m^2^)	0.970	0.949	0.992	0.007
	Uric Acid (mg/mL)	1.643	1.118	2.415	0.011

Abbreviation – HDL: high-density lipoprotein.

**Figure 2.**
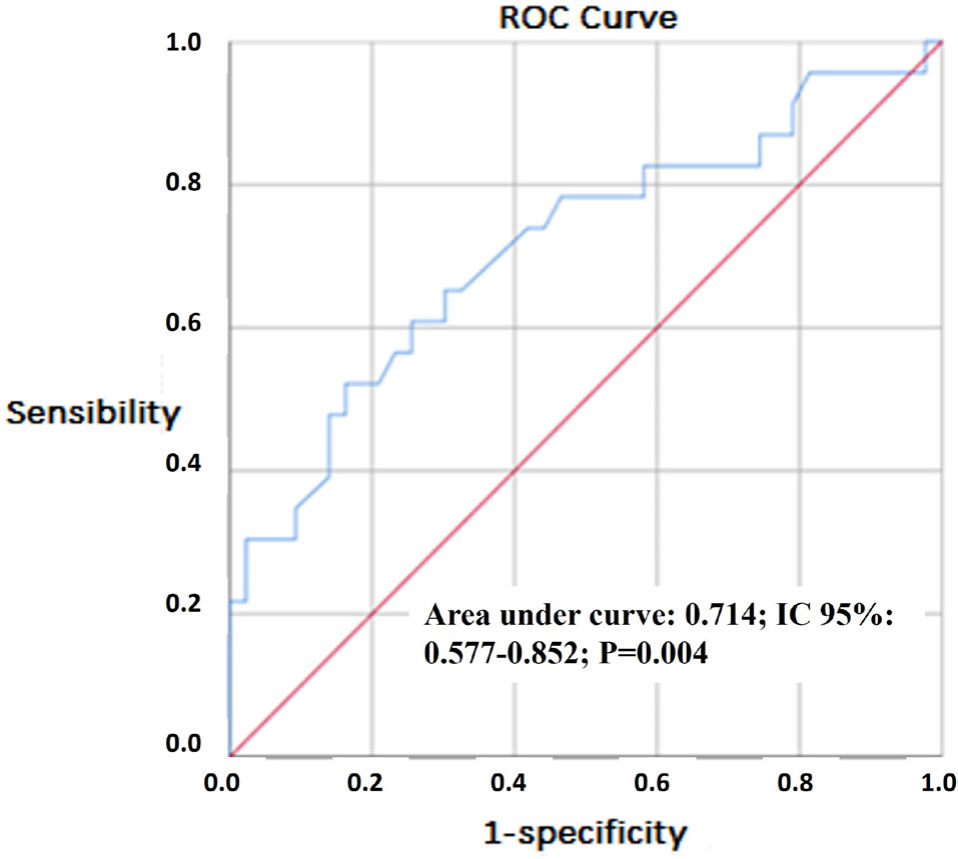
ROC curve of the total kidney length as a predictor for renal outcome.

**Figure 3.**
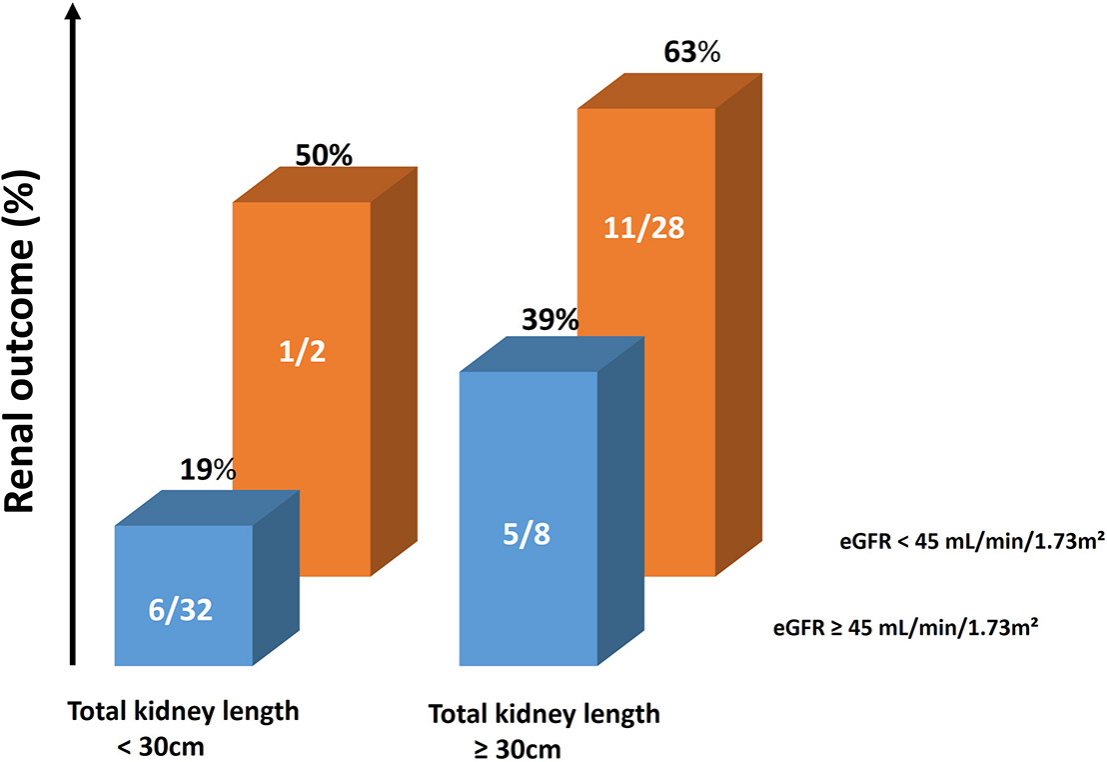
Probability of renal outcome according to total kidney length and eGFR.

Figure 2 shows the ROC curve, which evaluates the discriminatory power of the total kidney length in relation to the renal outcome. It can be observed that the area under the curve differs statistically from 0.5, which evaluates this power as statistically significant. At the cut-off point of >30 cm (according to the Youden index) the sensitivity of this sum was 65% and the specificity was 70%. The positive likelihood ratio (LR+) was 2.17 and the negative likelihood ratio (LR–) was 0.50. At the cut-off point of ≥ 36 cm, the sensitivity of this sum was 30% and the specificity was 98%, with LR+ of 15 and LR– of 0.71. At the cut-off point of ≥ 23 cm, the sensitivity of this sum was 96% and the specificity was 19%, with LR+ of 1.2 and LR– of 0.23. Figure 3 shows the absolute number and frequency of renal outcomes according to kidney length and eGFR. In this figure, it is possible to observe the influence of total kidney length, regardless of eGFR and eGFR regardless of total kidney length.

Nine patients died and among the causes of death, three were due to stroke, two due to cirrhosis and its complications, two due to sepsis and one due to an unknown cause. The clinical variables that differed between the subjects in whom the death outcome occurred and the other patients were presence of DM, coronary artery disease, cerebrovascular disease and atherosclerotic disease in any territory. The other clinical variables were homogeneous. Age was selected to be part of a multiple analysis because it was associated with death at the level of p = 0.081. These data are expressed in Table 4. Among the laboratory variables, none showed a statistically significant association with death. However, considering the eGFR (Deaths 59.5 ± 16.2 and non-deaths 78.4 ± 28.33), non-death was associated with death at the level of p = 0.056, this variable was included in multiple analyses.

**Table 4.** Clinical data of patients with ADPKD regarding the death outcome in a Brazilian cohort

	Death (n = 9)	Non-deaths (n = 61)	p
Age* (Years)	54 ± 14.6	44 ± 16.1	0.081
Non-white people (%)	0 (0%)	6 (10%)	0.657
Men (%)	6 (67%)	31 (51%)	0.374
Smoking^#^ (%)	7 (78%)	34 (56%)	0.235
*Diabetes mellitus*	6 (67%)	7 (11%)	<0.001
Weight (Kg)	81 ± 11.4	74 ± 15.1	0.205
Height (cm)	169 ± 4.6	168 ± 11	0.834
BMI (Kg/m²)	27.6 ± 4.61	25.4 ± 5.96	0.392
Presence of CAD	5 (56%)	3 (5%)	<0.001
Presence of CVD	6 (67%)	4 (7%)	<0.001
Presence of PAD	1 (11%)	5 (8%)	0.771
Atherosclerotic disease	6 (67%)	9 (15%)	<0.001
SBP (mmHg)	140 ± 9.1	134 ± 12.1	0.150
DBP (mmHg)	83 ± 7.4	84 ± 8.2	0.810
Left Kidney (cm)	14.3 ± 2.65	14.9 ± 3.18	0.641
Right Kidney (cm)	14.1 ± 2.59	14.8 ± 3.31	0.565
Total kidney length (cm)	28.4 ± 4.84	29.6 ± 6.07	0.576

Abbreviations – BMI: body mass index, CAD: coronary artery disease, CVD: cerebrovascular disease, PAD: peripheral arterial disease, SBP: systolic blood pressure, DAP: diastolic blood pressure. Notes – *At the beginning of the follow-up, ^#^active or previous.

The variables above, except for coronary artery disease and cerebrovascular disease due to their strong collinearity with the presence of atherosclerotic disease, were selected to compose multiple Cox analysis models. Using automatic variable selection (backward stepwise), the final model was obtained in which the presence of DM and eGFR were associated with the death outcome (Table 5). The presence of DM adjusted for the glomerular filtration rate was associated with the HR risk of death of 8.115, with 95% CI of 1.985–33.180, and each unit (mL/min/1.73 m^2^) more of eGFR was associated with HR (95% CI) of 0.957 (0.919–0.997), even after adjusting for the presence of DM.

**Table 5.** Multiple Cox analysis with the death outcome as an independent variable in a Brazilian cohort

		HR	95% CI		p
			inferior	Superior	
Step 1	*Diabetes mellitus*	6.252	1.091	35.834	0.040
	Age* (anos)	0.964	0.907	1.024	0.237
	Atherosclerotic disease	3.038	0.431	21.391	0.265
	CKD-EPI (mL/min/1.73 m^2^)	0.942	0.892	0.995	0.031
Step 2	*Diabetes mellitus*	9.994	2.136	46.758	0.003
	Age* (anos)	0.979	0.927	1.034	0.443
	CKD-EPI (mL/min/1.73 m^2^)	0.946	0.898	0.997	0.038
Step 3	*Diabetes mellitus*	8.115	1.985	33.170	0.004
	CKD-EPI (mL/min/1.73 m^2^)	0.957	0.919	0.997	0.033

Note – *At the beginning of the follow-up.

## Discussion

Several predictors of ADPKD progression are known. The present study aimed to identify, among a Brazilian single-center cohort, associations between clinical and laboratory variables with renal outcomes and mortality in ADPKD patients. We found that eGFR, total kidney length and serum uric acid level were independently associated with renal outcome. Furthermore, the presence of DM and eGFR were independent factors associated with mortality.

Renal outcome was associated with total kidney length measured by US and eGFR. It is known that ADPKD patients with larger kidneys start dialysis early^12,16,17^. A systematic review^18^ found that age and total renal volume were the indicators most frequently associated with ADPKD progression, followed by the estimated or measured glomerular filtration rate. Although most of these studies used the measurement of renal volume, both linear values of the largest renal axis and those of kidney volume (both assessed by US and magnetic resonance imaging) were associated with a faster chronic kidney disease (CKD) evolution^19^. In addition, it is important to note that our study used US measurements performed in the hospital clinical routine, which reflects that the simple renal dimension obtained in “real life” was able to predict prognosis. Buthani et al.^19^, mentioned above, pointed out that kidneys larger than the average of 16.5 cm have the best cut-off point to predict the development of stage 3 CKD, while our study showed a cut-off point for the renal outcome of 30 cm of the total kidney length i.e. approximately 15 cm in each kidney. Cornec-Le Gall and Le Meur^20^ argue against the value of kidney length to predict prognosis in ADPKD. Our data, however, favorably pointed to kidney length as a valid prognostic marker.

The increase in total kidney length can predict progression to the renal outcome even before the glomerular filtration rate falls. Apparently, glomerular filtration is maintained through the hyperfiltration of the remaining nephrons, and the measurement of eGFR can mask the true loss of function of the nephrons^21^.

Serum uric acid levels were also associated with renal outcome. There is evidence of an association of high levels of uric acid with the early onset of hypertension, greater renal volume, and increased risk for ESKD in ADPKD patients regardless of gender, body mass index and renal function^22^. It has been described that greater serum uric acid levels are a risk factor for endothelial dysfunction in ADPKD patients even in early stages^23^. Uric acid may be associated with an increase in proinflammatory mediators, such as tumor necrosis factor (TNF-α), chemokines^24^ and CRP^25^, which can lead to renal parenchyma fibrosis and progression of kidney disease. Uric acid impairs nitric oxide synthesis in cultured endothelial cells^26,27^, and is associated with increased pro-oxidative activity that can contribute to endothelial dysfunction^28–30^. In ADPKD, endothelial dysfunction can lead to increased renal vascular resistance and a consequent decrease in renal blood flow that precedes the decline of glomerular filtration rate, and can, therefore predict the progression of renal disease even at normal glomerular filtration levels^31^. Since uric acid elevation is common in metabolic syndrome, and obesity and metabolic syndrome are associated with a progression of ADPKD^32,33^, it is a pertinent idea that metabolic syndrome could be explained, at least in part, by the association between uric acid and outcome in our study.

Reed et al.^34^ found that DM and eGFR were independently associated with death. Patients with ADPKD and type II DM have higher renal volumes, earlier diagnosis of hypertension and may die at a younger age compared to those patients with isolated polycystic kidney disease^34^. Cardiovascular complications are the main causes of death in ADPKD, as observed in DM patients^35,36^. Although Patch et al.^37^ did not target DM as a prognostic factor, they found that DM was identified as a prognostic marker, and mortality was significantly higher in patients with polycystic kidney disease who were diabetics^37^. Possibly, in ADPKD patients, even with normal renal function, there is a compromise in the function of pancreatic beta cells, promoting abnormal insulin secretion^38^. In addition, these patients probably have a marked reduction in insulin sensitivity^39^, which may be due to abnormalities in the membrane and cytoskeleton that occur in the disease^40^. Although, Pietrzak-Nowacka et al.^38^ did not find insulin resistance in their work. Therefore, this last affirmation is not a consensus in the literature yet^38^.

It is necessary to recognize some limitations of the present study such as the small sample size, although the analyzed sample was sufficient to identify factors measured in the clinical routine as predictors of the outcomes in ADPKD patients^41^. Magnetic resonance was not available at the time of diagnosis of our patients for more accurate measurement of total kidney length, however we identified that the US measurement has a prognostic value, which is easy to access in health services. The calculation of renal volume by the ellipsoid equation was not used in this study, as we did not have complete data on renal thickness and width, since the tests used in this study were not done specifically for this work. However, our study identifies that the measurement of total kidney length in routine clinical examinations is able to predict the prognosis of patients. In addition, we do not have a genetic diagnosis of ADPKD to assess the prognostic value of different mutations. However, this analysis is unusual in clinical practice since few facilities in developing countries have access to this resource. Finally, we were not sure about family history of all patients, but when we did not have family information about a patient, we included these patients only if they had more than 20 cysts and kidney length more than 13 cm, according to Iliuta et al^14^.

As a strong point, we were able to identify that clinical and laboratory data of ADPKD patients from a Brazilian cohort were associated with the progression of the renal disease. We found an independent association of total kidney length, glomerular filtration rate and serum uric acid levels with the progression to renal outcomes. In addition, there was an independent association between the presence of diabetes mellitus and the glomerular filtration rate with mortality.

In conclusion, this longitudinal study identified associations between clinical and laboratory variables with renal outcomes and mortality in ADPKD patients. These markers can easily help to predict the progression of this disease, indicating the need for an earlier and a closer follow up. In addition, these findings corroborate the hypothesis that such factors are also important prognostic predictors in a Brazilian cohort.

## References

[1] United States Renal Data System. (2019). US Renal Data System 2019 Annual Data Report: epidemiology of kidney
disease in the United States..

[2] Perrone RD, Malek AM, Watnick T (2015). Vascular complications in autosomal dominant polycystic kidney
disease.. Nat Rev Nephrol..

[3] Ecder T, Schrier RW (2001). Hypertension in autosomal-dominant polycystic kidney disease:
early occurrence and unique aspects.. J Am Soc Nephrol..

[4] Perrone RD, Oberdhan D, Ouyang J, Bichet DG, Budde K, Chapman AB (2023). OVERTURE: a worldwide, prospective, observational study of
disease characteristics in patients with ADPKD.. Kidney Int Rep..

[5] Cornec-Le Gall E, Audrézet MP, Rousseau A, Hourmant M, Renaudineau E, Charasse C (2016). The PROPKD Score: a new algorithm to predict renal survival in
autosomal dominant polycystic kidney disease.. J Am Soc Nephrol..

[6] Irazabal MV, Rangel LJ, Bergstralh EJ, Osborn SL, Harmon AJ, Sundsbak JL (2015). Imaging classification of autosomal dominant polycystic kidney
disease: a simple model for selecting patients for clinical
trials.. J Am Soc Nephrol..

[7] Corradi V, Gastaldon F, Caprara C, Giuliani A, Martino F, Ferrari F (2017). Predictors of rapid disease progression in autosomal dominant
polycystic kidney disease.. Minerva Med..

[8] Uchiyama K, Mochizuki T, Shimada Y, Nishio S, Kataoka H, Mitobe M (2021). Factors predicting decline in renal function and kidney volume
growth in autosomal dominant polycystic kidney disease: a prospective cohort
study (Japanese Polycystic Kidney Disease registry: J-PKD).. Clin Exp Nephrol..

[9] Torres VE (2000). Hypertension, proteinuria, and progression of autosomal dominant
polycystic kidney disease: where do we go from here?. Am J Kidney Dis..

[10] Ecder T, Chapman AB, Brosnaham GM, Edelstein CL, Johnson AM, Schrier RW (2000). Effect of antihypertensive therapy on renal function and urinary
albumin excretion in hypertensive patients with autosomal dominant
polycystic kidney disease.. Am J Kidney Dis..

[11] Klahr S, Breyer J, Beck G, Dennis V, Hartman J, Roth D (1995). Dietary protein restriction, blood pressure control, and the
progression of polycystic kidney disease.. J Am Soc Nephrol..

[12] Pei Y, Hwang YH, Conklin J, Sundsbak JL, Heyer CM, Chan W (2015). Imaging-based diagnosis of autosomal dominant polycystic kidney
disease.. J Am Soc Nephrol..

[13] Pei Y, Obaji J, Dupuis A, Paterson AD, Magistroni R, Dicks E (2009). Unified criteria for ultrasonographic diagnosis of
ADPKD.. J Am Soc Nephrol..

[14] Iliuta IA, Kalatharan V, Wang K, Cornec-Le Gall E, Conklin J, Pourafkari M (2017). Polycystic Kidney Disease without an Apparent Family
History.. J Am Soc Nephrol..

[15] Coresh J, Turin TC, Matsushita K, Sang Y, Ballew SH, Appel LJ (2014). Decline in estimated glomerular filtration rate and subsequent
risk of end-stage renal disease and mortality.. JAMA..

[16] Nicolau C, Torra R, Bianchi L, Vilana R, Gilabert R, Darnell A (2000). Abdominal sonographic study of autosomal dominant polycystic
kidney disease.. J Clin Ultrasound..

[17] Granthan JJ, Torres VE, Chapman AB, Guay-Woodford LM, Bae KT, King BF (2006). Volume progression in polycystic kidney disease.. N Engl J Med..

[18] Woon C, Bielinski-Bradbury A, O’Reilly K, Robinson P (2015). A systematic review of the predictors of disease progression in
patients with autosomal dominant polycystic kidney disease.. BMC Nephrol..

[19] Bhutani H, Smith V, Rahbari-Oskoui F, Mittal A, Grantham JJ, Torres VE (2015). A comparison of ultrasound and magnetic resonance imaging shows
that kidney length predicts chronic kidney disease in autosomal dominant
polycystic kidney disease.. Kidney Int..

[20] Cornec-Le Gall E, Le Meur Y (2015). Can ultrasound kidney length qualify as an early predictor of
progression to renal insufficiency in autosomal dominant polycystic kidney
disease?. Kidney Int..

[21] Grantham JJ, Torres VE (2016). The importance of total kidney volume in evaluating progression
of polycystic kidney disease.. Nat Rev Nephrol..

[22] Helal I, McFann K, Reed B, Yan XD, Schrier RW, Fick-Brosnahan GM (2013). Serum uric acid, kidney volume and progression in
autosomal-dominant polycystic kidney disease.. Nephrol Dial Transplant..

[23] Kocyigit I, Yilmaz MI, Orscelik O, Sipahioglu MH, Unal A, Eroglu E (2013). Serum uric acid levels and endothelial dysfunction in patients
with autosomal dominant polycystic kidney disease.. Nephron Clin Pract..

[24] Zhou Y, Fang L, Jiang L, Wen P, Cao H, He W (2012). Uric acid induces renal inflammation via activating tubular NF-κB
signaling pathway.. PLoS One..

[25] Kang DH, Park SK, Lee IK, Johnson RJ (2005). Uric acid-induced C-reactive protein expression: implication on
cell proliferation and nitric oxide production of human vascular
cells.. J Am Soc Nephrol..

[26] Khosla UM, Zharikov S, Finch JL, Nakagawa T, Roncal C, Mu W (2005). Hyperuricemia induces endothelial dysfunction.. Kidney Int..

[27] Mercuro G, Vitale C, Cerquetani E, Zoncu S, Deidda M, Fini M (2004). Effect of hyperuricemia upon endothelial function in patients at
increased cardiovascular risk.. Am J Cardiol..

[28] Zharikov S, Krotova K, Hu H, Baylis C, Johnson RJ, Block ER (2008). Uric acid decreases NO production and increases arginase activity
in cultured pulmonary artery endothelial cells.. Am J Physiol Cell Physiol..

[29] Sánchez-Lozada LG, Soto V, Tapia E, Avila-Casado C, Sautin YY, Nakagawa T (2008). Role of oxidative stress in the renal abnormalities induced by
experimental hyperuricemia.. Am J Physiol Renal Physiol..

[30] Sánchez-Lozada LG, Tapia E, López-Molina R, Nepomuceno T, Soto V, Avila-Casado C (2007). Effects of acute and chronic L-arginine treatment in experimental
hyperuricemia.. Am J Physiol Renal Physiol..

[31] Torres VE, King BF, Chapman AB, Brummer ME, Bae KT, Glockner JF (2007). Magnetic resonance measurements of renal blood flow and disease
progression in autosomal dominant polycystic kidney disease.. Clin J Am Soc Nephrol..

[32] Nowak KL, You Z, Gitomer B, Brosnahan G, Torres VE, Chapman AB (2018). Overweight and obesity are predictors of progression in early
autosomal dominant polycystic kidney disease.. J Am Soc Nephrol..

[33] Nowak KL, Hopp K (2020). Metabolic reprogramming in autosomal dominant polycystic kidney
disease: evidence and therapeutic potential.. Clin J Am Soc Nephrol..

[34] Reed B, Helal I, McFann K, Wang W, Yan XD, Schrier RW (2012). The impact of type II diabetes mellitus in patients with
autosomal dominant polycystic kidney disease.. Nephrol Dial Transplant..

[35] Fick GM, Johnson AM, Hammond WS, Gabow PA (1995). Causes of death in autosomal dominant polycystic kidney
disease.. J Am Soc Nephrol..

[36] Perrone RD, Ruthazer R, Terrin NC (2001). Survival after end-stage renal disease in autosomal dominant
polycystic kidney disease: contribution of extrarenal complications to
mortality.. Am J Kidney Dis..

[37] Patch C, Charlton J, Roderick PJ, Gulliford MC (2011). Use of antihypertensive medications and mortality of patients
with autosomal dominant polycystic kidney disease: a population-based
study.. Am J Kidney Dis..

[38] Pietrzak-Nowacka M, Safranow K, Byra E, Nowosiad M, Marchelek-Mys´liwiec M, Ciechanowski K (2010). Glucose metabolism parameters during an oral glucose tolerance
test in patients with autosomal dominant polycystic kidney
disease.. Scand J Clin Lab Invest..

[39] Vareesangthip K, Tong P, Wilkinson R, Thomas TH (1997). Insulin resistance in adult polycystic kidney
disease.. Kidney Int..

[40] Vareesangthip K, Thomas TH, Tong P, Wilkinson R (1996). Abnormal erythrocyte membrane fluidity in adult polycystie kidney
disease: difference between intact cells and ghost
membranes.. Eur J Clin Invest..

[41] Vittinghoff E, McCulloch CE (2007). Relaxing the rule of ten events per variable in logistic and Cox
regression.. Am J Epidemiol..

