# Supplementary material for: Predictors of autosomal dominant polycystic kidney disease progression: a Brazilian single-center cohort
Source: J Bras Nefrol. 2024 Jun 24;46(3):e20230040. doi: 10.1590/2175-8239-JBN-2023-0040en (PMC11210993; doi:10.1590/2175-8239-JBN-2023-0040en)
Supplement: Translation

# Translation: Preditores de progressão da doença renal policística autossômica dominante: uma coorte brasileira de centro único

**DOI:** 10.1590/2175-8239-JBN-2023-0040pt

**Authors:** Igor Hitoshi Nishimoto, Andrey Gonçalves Santos, Júlia Mandelbaun Bianchini, Luiz Gustavo Brenneisen Santos, Maria Carolina Rodrigues Martini, Vanessa dos Santos Silva, Luis Cuadrado Martin

**Affiliations:** 1 Universidade Estadual Paulista "Júlio de Mesquita Filho", Escola de Medicina, Botucatu, SP, Brasil.; 2 Universidade Estadual Paulista "Júlio de Mesquita Filho", Escola de Medicina, Departamento de Medicina Interna, Botucatu, SP, Brasil.

**Keywords:** Rim Policístico Autossômico Dominante, Insuficiência Renal, Taxa, Mortalidade, Fatores de Risco

## Abstract

**Introdução:**

É importante identificar fatores de risco para progressão da doença renal policística autossômica dominante (DRPAD). Entretanto, são escassos os estudos que avaliam esse assunto utilizando amostra brasileira. Portanto, o objetivo deste estudo foi identificar fatores de risco para desfechos renais e óbito em coorte brasileira de pacientes com DRPAD.

**Métodos:**

Os pacientes tiveram o primeiro atendimento médico entre janeiro/2002 e dezembro/2014, sendo acompanhados até dezembro/2019. Associações entre variáveis clínicas e laboratoriais com desfecho primário (redução sustentada de pelo menos 57% na TFGe em relação ao valor basal, necessidade de diálise ou transplante renal) e desfecho secundário (óbito por qualquer causa) foram analisadas pelo modelo de regressão múltipla de Cox. Entre 80 pacientes com DRPAD, foram excluídos aqueles menores de 18 anos, com TFG <30 mL/min/1,73 m2 e/ou aqueles com dados ausentes. Foram acompanhados 70 pacientes.

**Resultados:**

Fatores independentemente associados aos desfechos renais foram: comprimento renal total – Razão de Risco (HR) ajustada com intervalo de confiança de 95% (IC 95%): 1,137 (1,057–1,224), taxa de filtração glomerular – HR (IC 95%): 0,970 (0,949–0,992) e nível sérico de ácido úrico - HR (IC 95%): 1,643 (1,118–2,415). Diabetes mellitus – HR (IC 95%): 8,115 (1,985–33,180) e TFG – HR (IC 95%): 0,957 (0,919–0,997) foram associados ao desfecho secundário.

**Conclusões:**

Esses achados corroboram a hipótese de que comprimento renal total, TFG e nível sérico de ácido úrico podem ser importantes preditores prognósticos de DRPAD em uma coorte brasileira, o que pode ajudar a selecionar pacientes que necessitam de acompanhamento mais próximo.

## Introdução

A doença renal policística autossômica dominante (DRPAD), a causa monogênica mais comum de doença renal em estágio terminal (DRET), é caracterizada pelo desenvolvimento inexorável de cistos renais, hipertensão e destruição do parênquima renal^1^. Essa doença é caracterizada pela formação de múltiplos cistos nos rins, cujo crescimento leva à compressão e isquemia dos néfrons adjacentes e a um processo inflamatório que resulta em fibrose e comprometimento progressivo da função renal.

As principais causas de óbito em pacientes com DRPAD são as doenças cardiovasculares^2^. A pressão arterial elevada está presente em mais da metade dos pacientes antes do declínio da taxa de filtração glomerular^3^ e é o principal determinante desse desfecho. O mau prognóstico dos pacientes com DRPAD está relacionado ao tamanho maior dos rins, ao sexo masculino, à hipertensão mal tratada e ao gene PKD1^4–6^. Pacientes negros e aqueles com hematúria antes dos 30 anos de idade, início de hipertensão antes dos 35 anos, proteinúria e hiperlipidemia também apresentam maior probabilidade de ter um pior desfecho^4,7^.

Além disso, baixos níveis de lipoproteína de alta densidade (HDL) e níveis elevados de colesterol e lipoproteína de baixa densidade (LDL) foram identificados como fatores de risco para a progressão da DRPAD^8–11^.

Em pacientes com DRPAD, a taxa de filtração glomerular diminui ao longo de 10 a 20 anos a partir do diagnóstico, e cerca de 60% progridem para DRET até a sétima década de vida^8^. O tratamento da DRPAD é direcionado principalmente aos sintomas e complicações.

Diante desses pontos, é extremamente importante identificar os preditores de progressão da DRPAD, a fim de acompanhar de perto pacientes com maior risco e, ao mesmo tempo, mitigar o agravamento da doença e suas complicações. No entanto, ainda não foram identificados estudos que tenham avaliado esse assunto dentro de uma coorte brasileira.

Assim, este estudo tem como objetivo identificar fatores de risco, buscando associações entre variáveis clínicas e laboratoriais com desfechos renais e óbito, em pacientes com DRPAD acompanhados em uma coorte brasileira de centro único.

## Métodos

Um estudo longitudinal foi realizado em uma coorte de pacientes com DRPAD, o qual foi aprovado pelo comitê de ética local sob o número: 3.383.261. Foram consultados os prontuários de todos os pacientes que tiveram seu primeiro atendimento médico no Serviço de Nefrologia da Faculdade de Medicina do Hospital das Clínicas de Botucatu de janeiro de 2002 a dezembro de 2014, em busca de pacientes com DRPAD. Isso foi feito por meio de uma busca ativa de todos os exames de imagem nos prontuários médicos. Foram avaliadas ultrassonografia (US) de abdome total, US renal e tomografia computadorizada (TC) abdominal. Esses exames foram realizados de acordo com a rotina hospitalar sem qualquer padronização específica, uma vez que este é um estudo de vida real.

O diagnóstico de DRPAD^12,13^ foi considerado:

- Para indivíduos pertencentes a famílias acometidas pela DRPAD: presença de três ou mais cistos, unilaterais ou bilaterais, em pacientes com idade entre 15 e 39 anos; dois ou mais cistos em cada rim em pacientes com idade entre 40 e 59 anos e quatro ou mais cistos em cada rim em pacientes com idade superior a 60 anos;
- Em indivíduos com suspeita de DRPAD, mas sem histórico familiar positivo: presença de 20 ou mais cistos em cada rim, especialmente se os rins estiverem aumentados ou cistos extra-renais, e na ausência de características evidentes de outras doenças císticas^14^.

Foram incluídas no estudo pessoas com DRPAD de acordo com os critérios acima e com idade superior a 18 anos. Foram excluídos pacientes com taxa de filtração glomerular estimada (TFGe) <30 mL/min/1,73 m^2^, segundo a equação CKD-EPI, no início do acompanhamento, bem como pacientes com dados incompletos.

Os pacientes foram acompanhados até dezembro de 2019. O desfecho primário foi a redução sustentada de pelo menos 57% na TFGe em relação ao valor basal (essa redução é equivalente ao dobro da creatinina, que é um desfecho renal clássico)^15^ e necessidade de diálise ou transplante renal. O desfecho secundário foi óbito por qualquer causa. As variáveis independentes foram idade, sexo, raça, a soma do maior eixo renal (comprimento renal total), tabagismo, peso, altura, índice de massa corporal, presença de diabetes mellitus (DM), presença de doença arterial coronariana, presença de doença cerebrovascular, presença de doença arterial periférica e presença de doença aterosclerótica (doença arterial coronariana, doença cerebrovascular ou doença arterial periférica), todas essas variáveis no início do estudo. Os valores de pressão arterial sistólica e diastólica foram considerados como a média de todos os registros disponíveis. Os seguintes dados laboratoriais foram avaliados no início do estudo: creatinina sérica, taxa de filtração glomerular estimada (TFGe), níveis séricos de potássio, cálcio, fósforo, sódio, colesterol total, HDL, LDL, triglicérides, paratormônio, proteína C reativa e nível sérico de ácido úrico. Também foram avaliados hemoglobina, leucócitos, plaquetas, volume urinário, proteinúria, densidade urinária, presença de hematúria macroscópica e sódio urinário de 24 horas.

As variáveis categóricas foram analisadas de acordo com o teste qui-quadrado; as variáveis contínuas foram analisadas usando o teste t de Student se houvesse distribuição normal e teste de Mann-Whitney quando os pacientes não apresentavam distribuição normal. Os resultados foram listados em tabelas utilizando valores de média e desvio padrão ou frequência absoluta e relativa. As variáveis que foram associadas aos desfechos no nível de p < 0,10 foram incluídas no modelo de regressão múltipla de Cox. As colinearidades foram testadas e, quando presentes, foi escolhida a variável com maior significância clínica. Em seguida, foi utilizada a seleção automática de variáveis (*stepwise backward*). Também foi utilizada uma análise da curva ROC (*Receiver Operating Characteristic Curve*) para avaliar o poder discriminatório do comprimento renal total em relação ao desfecho renal. O índice de Youden (maior soma de especificidade e sensibilidade) foi usado para verificar o melhor ponto de corte e as razões de verossimilhança positiva e negativa também foram calculadas. Os resultados foram discutidos ao nível de p < 0,05.

## Resultados

Um total de 1.761 prontuários médicos foram consultados para buscar pacientes com DRPAD. Após análise de todos os prontuários, foram encontrados 156 pacientes com cistos renais. A partir da exclusão de pacientes com cistos simples e outras doenças renais císticas que não a DRPAD, o número obtido de pacientes com DRPAD foi de 80. De acordo com os critérios de exclusão, foram excluídos seis pacientes com menos de 18 anos e quatro com TFGe <30 mL/min/1,73 m^2^ no início do acompanhamento (Figura 1).

**Figura 1.** Fluxograma de inclusão do paciente.

O estudo de coorte foi composto por 70 pacientes, com uma média de idade de 46 ± 16,1 anos, 37 homens (53%) e 6 não brancos (9%). Houve 65 pacientes submetidos a ultrassonografia e 5 pacientes submetidos a exames de TC. A maioria era de fumantes ativos ou inativos (57%), 19% eram diabéticos e 21% apresentavam alguma doença aterosclerótica. O período de acompanhamento variou de 1,2 a 198 meses, com média de 109 ± 55 meses e mediana de 110 (intervalo interquartil: 71–158) meses.

O desfecho renal foi observado em 23 pacientes. O comprimento renal total foi estatisticamente diferente entre progressores e não progressores (Tabela 1). Entre as variáveis laboratoriais, a creatinina sérica, TFGe com base na creatinina sérica, HDL, nível sérico de ácido úrico e densidade urinária foram associados ao desfecho primário (renal) (Tabela 2).

**Tabela 1.** Dados clínicos de pacientes com DRPAD em relação aos desfechos renais (duplicação da creatinina ou entrada em diálise) em uma coorte brasileira

	Desfecho renal (n = 23)	Sem desfecho renal (n = 47)	p
Idade* (Anos)	47 ± 11,6	45 ± 18,1	0,532
Pessoas não brancas (%)	2 (9%)	4 (9%)	0,980
Homens (%)	2 (9%)	4 (9%)	0,980
Tabagismo^#^ (%)	16 (70%)	24 (51%)	0,234
Diabetes mellitus	7 (30%)	6 (13%)	0,098
Peso (Kg)	76 ± 15,6	74 ± 14,5	0,691
Altura (cm)	169 ± 11,2	167 ± 9,3	0,477
IMC (Kg/m²)	26,93 ± 4,16	25,08 ± 6,37	0,325
Presença de DAC	3 (13%)	5 (11%)	0,766
Presença de DCBV	2 (9%)	4 (9%)	0,467
Presença de DAP	2 (9%)	4 (9%)	0,979
Doença aterosclerótica	5 (22%)	10 (21%)	0,964
PAS (mmHg)	137 ± 12,4	133 ± 11,5	0,180
PAD (mmHg)	84 ± 7,9	83 ± 8,1	0,148
Rim Esquerdo (cm)	16,3 ± 3,61	14,0 ± 2,46	0,003
Rim Direito (cm)	16,3 ± 3,39	13,9 ± 2,79	0,003
Comprimento renal total (cm)	32,6 ± 6,62	27,8 ± 4,76	0,001

Abreviações – IMC: índice de massa corporal, DAC: doença arterial coronariana, DCBV: doença cerebrovascular, DAP: doença arterial periférica, PAS: pressão arterial sistólica, PAD: pressão arterial diastólica. Notas – *No início do acompanhamento, ^#^ativo ou anterior.

**Tabela 2.** Dados laboratoriais de pacientes com DRPAD relativos a desfechos renais (duplicação da creatinina ou entrada em diálise) em uma coorte brasileira

	Desfecho renal (n = 23)	Sem desfecho renal (n = 47)	p
Creatinina (mg/dL)	1,4 ± 0,43	1,0 ± 0,27	<0,001
CKD-EPI (ml/min/1,73m²)	61,1 ± 26,03	83,3 ± 25,77	<0,001
Potássio (mEq/L)	4,5 ± 0,56	4,4 ± 0,57	0,311
Cálcio (mg/dL)	9,2 ± 0,75	9,5 ± 0,69	0,082
Fósforo (mg/dL)	3,7 ± 0,57	3,6 ± 0,66	0,602
Sódio (mmol/L)	141,4 ± 1,75	141,2 ± 2,75	0,692
PTH (pg/mL)	88,3 ± 46,68	67,2 ± 54,11	0,141
Hemoglobina (g/dL)	13,3 ± 1,90	13,6 ± 1,58	0,602
Plaquetas (10³/mm³)	266 ± 116,3	237 ± 72,2	0,218
Leucócitos (10^3^/mm^3^)	8,9 ± 5,50	7,7 ± 2,24	0,234
PCR (mg/dL)	1,1± 1,75	1,1 ± 1,12	0,955
Colesterol total (mg/dL)	179,2 ± 36,85	176,2 ± 33,82	0,737
Triglicérides (mg/dL)	168,6 ± 61,58	141,0 ± 79,35	0,147
HDL (mg/dL)	39,4 ± 9,84	46,8 ± 10,47	0,006
LDL calculado (mg/dL)	106,1 ± 32,72	101,3 ± 26,25	0,509
Proteinúria (g/24h)	0,04 ± 0,065	0,06 ± 0,173	0,686
Ácido Úrico (mg/mL)	6,7 ± 1,06	5,8 ± 1,40	0,008
Volume urinário (mL)	1929 ± 558,9	1742 ± 725,5	0,324
Densidade urinária (g/dL)	1011,2 ± 1,77	1013,9 ± 4,09	0,004
RBC/HPF	6,0 ± 10,94	5,0 ± 11,78	0,729
Sódio Urinário (mEq/24h)	147,1 ± 69,55	197,5 ± 87,55	0,303

Abreviações – PTH: paratormônio, PCR: Proteína C-reativa, HDL: proteína de alta densidade; LDL: proteína de baixa densidade, WBC: leucócitos, RBC/HPF: hemácias por campo de grande aumento.

As variáveis acima foram selecionadas para modelos de regressão múltipla de Cox (exceto a creatinina sérica, por apresentar uma forte colinearidade com a taxa de filtração glomerular). A presença de DM também foi selecionada para compor a análise múltipla. Usando a seleção *stepwise backward*, obteve-se o modelo final no qual há uma associação entre desfecho renal e comprimento renal total, TFGe e nível sérico de ácido úrico (Tabela 3). No modelo final ajustado, cada centímetro no comprimento renal total foi associado a uma Razão de Risco (HR, do inglês *Hazard Ratio*) de 1,137 para o desfecho renal, com um Intervalo de Confiança de 95% (IC 95%) de 1,057–1,224; para cada unidade (mL/min/1,73 m^2^) a mais de taxa de filtração glomerular, obteve-se uma HR (IC 95%) de 0,970 (0,949–0,992) e cada unidade (mg/mL) de nível sérico de ácido úrico foi associada a uma HR (IC 95%) de 1,643 (1,118-2,415).

**Tabela 3.** Análise múltipla de Cox com desfecho renal como variável independente em uma coorte brasileira

		HR	IC 95%		p
			Inferior	Superior	
Etapa 1	Comprimento renal total (cm)	1,123	1,043	1,209	0,002
	CKD-EPI (mL/min/1,73 m^2^)	0,977	0,955	0,999	0,045
	Ácido Úrico (mg/mL)	1,555	0,996	2,426	0,052
	Diabetes mellitus	1,175	0,448	3,084	0,743
	HDL (mg/dL)	0,966	0,906	1,031	0,298
	Densidade urinária (g/dL)	0,889	0,730	1,083	0,244
Etapa 2	Comprimento renal total (cm)	1,122	1,043	1,207	0,002
	CKD-EPI (mL/min/1,73 m^2^)	0,977	0,955	1,000	0,048
	Ácido Úrico (mg/mL)	1,550	0,996	2,413	0,052
	HDL (mg/dL)	0,963	0,906	1,024	0,234
	Densidade urinária (g/dL)	0,889	0,730	1,083	0,244
Etapa 3	Comprimento renal total (cm)	1,123	1,043	1,210	0,002
	CKD-EPI (mL/min/1,73 m^2^)	0,972	0,952	0,993	0,009
	Ácido Úrico (mg/mL)	1,443	0,947	2,198	0,088
	HDL (mg/dL)	0,963	0,909	1,020	0,195
Etapa 4	Comprimento renal total (cm)	1,137	1,057	1,224	0,001
	CKD-EPI (mL/min/1,73 m^2^)	0,970	0,949	0,992	0,007
	Ácido Úrico (mg/mL)	1,643	1,118	2,415	0,011

Abreviação – HDL: lipoproteína de alta densidade.

A Figura 2 mostra a curva ROC, que avalia o poder discriminatório do comprimento renal total em relação ao desfecho renal. Observa-se que a área sob a curva difere estatisticamente de 0,5, o que avalia esse poder como estatisticamente significativo. No ponto de corte de ^≥^30 cm (de acordo com o índice de Youden), a sensibilidade dessa soma foi de 65% e a especificidade foi de 70%. A razão de verossimilhança positiva (RV+) foi de 2,17 e a razão de verossimilhança negativa (RV–) foi de 0,50. No ponto de corte de ^≥^36 cm, a sensibilidade dessa soma foi de 30% e a especificidade foi de 98%, com RV+ de 15 e RV– de 0,71. No ponto de corte de > 23 cm, a sensibilidade dessa soma foi de 96% e a especificidade foi de 19%, com RV+ de 1,2 e RV– de 0,23. A Figura 3 mostra o número absoluto e a frequência dos desfechos renais de acordo com o comprimento renal e a TFGe. Nessa figura, é possível observar a influência do comprimento renal total, independentemente da TFGe, e da TFGe, independentemente do comprimento renal total.

**Figura 2.** Curva ROC do comprimento renal total como preditor de desfecho renal.

**Figura 3.** Probabilidade de desfecho renal de acordo com o comprimento renal total e TFGe.

Nove pacientes foram a óbito e, entre as causas de óbito, três foram por acidente vascular cerebral, dois devido à cirrose e suas complicações, dois devido à sepse e um devido a uma causa desconhecida. As variáveis clínicas que diferiram entre os indivíduos em que ocorreu o desfecho de óbito e os demais pacientes foram a presença de DM, doença arterial coronariana, doença cerebrovascular e doença aterosclerótica em qualquer território. As demais variáveis clínicas foram homogêneas. A idade foi selecionada para fazer parte de uma análise múltipla porque esteve associada ao óbito no nível de p = 0,081. Esses dados estão expressos na Tabela 4. Entre as variáveis laboratoriais, nenhuma apresentou uma associação estatisticamente significativa com óbito. No entanto, considerando a TFGe (óbitos 59,5 ± 16,2 e não óbitos 78,4 ± 28,33), o não óbito foi associado ao óbito no nível de p = 0,056, e essa variável foi incluída em múltiplas análises.

**Tabela 4.** Dados clínicos de pacientes com DRPAD em relação ao desfecho óbito em uma coorte brasileira

	Óbito (n = 9)	Não óbito (n = 61)	p
Idade* (Anos)	54 ± 14,6	44 ± 16,1	0,081
Pessoas não brancas (%)	0 (0%)	6 (10%)	0,657
Homens (%)	6 (67%)	31 (51%)	0,374
Tabagismo^#^ (%)	7 (78%)	34 (56%)	0,235
Diabetes mellitus	6 (67%)	7 (11%)	<0,001
Peso (Kg)	81 ± 11,4	74 ± 15,1	0,205
Altura (cm)	169 ± 4,6	168 ± 11	0,834
IMC (Kg/m²)	27,6 ± 4,61	25,4 ± 5,96	0,392
Presença de DAC	5 (56%)	3 (5%)	<0,001
Presença de DCBV	6 (67%)	4 (7%)	<0,001
Presença de DAP	1 (11%)	5 (8%)	0,771
Doença aterosclerótica	6 (67%)	9 (15%)	<0,001
PAS (mmHg)	140 ± 9,1	134 ± 12,1	0,150
PAD (mmHg)	83 ± 7,4	84 ± 8,2	0,810
Rim Esquerdo (cm)	14,3 ± 2,65	14,9 ± 3,18	0,641
Rim Direito (cm)	14,1 ± 2,59	14,8 ± 3,31	0,565
Comprimento renal total (cm)	28,4 ± 4,84	29,6 ± 6,07	0,576

Abreviações – IMC: índice de massa corporal, DAC: doença arterial coronariana, DCBV: doença cerebrovascular, DAP: doença arterial periférica, PAS: pressão arterial sistólica, PAD: pressão arterial diastólica. Notas – *No início do acompanhamento, ^#^ativo ou anterior.

As variáveis acima, exceto doença arterial coronariana e doença cerebrovascular devido à sua forte colinearidade com a presença de doença aterosclerótica, foram selecionadas para compor modelos de análise múltipla de Cox. Utilizando a seleção automática de variáveis (*stepwise backward*), obteve-se o modelo final no qual a presença de DM e TFGe foram associadas ao desfecho de óbito (Tabela 5). A presença de DM ajustada para a taxa de filtração glomerular foi associada a uma HR de risco de óbito de 8,115, com IC de 95% de 1,985-33,180, e cada unidade (mL/min/1,73 m^2^) a mais de TFGe foi associada a uma HR (IC 95%) de 0,957 (0,919–0,997), mesmo após o ajuste para a presença de DM.

**Tabela 5.** Análise múltipla de Cox com o desfecho óbito como variável independente em uma coorte brasileira

		HR	IC 95%		p
			Inferior	Superior	
Etapa 1	Diabetes mellitus	6,252	1,091	35,834	0,040
	Idade* (anos)	0,964	0,907	1,024	0,237
	Doença aterosclerótica	3,038	0,431	21,391	0,265
	CKD-EPI (mL/min/1,73 m^2^)	0,942	0,892	0,995	0,031
Etapa 2	Diabetes mellitus	9,994	2,136	46,758	0,003
	Idade* (anos)	0,979	0,927	1,034	0,443
	CKD-EPI (mL/min/1,73 m^2^)	0,946	0,898	0,997	0,038
Etapa 3	Diabetes mellitus	8,115	1,985	33,170	0,004
	CKD-EPI (mL/min/1,73 m^2^)	0,957	0,919	0,997	0,033

Nota – *No início do acompanhamento.

## Discussão

São conhecidos diversos preditores de progressão da DRPAD. O presente estudo teve como objetivo identificar, em uma coorte brasileira de centro único, associações entre variáveis clínicas e laboratoriais com desfechos renais e mortalidade em pacientes com DRPAD. Descobrimos que a TFGe, o comprimento renal total e o nível sérico de ácido úrico foram independentemente associados ao desfecho renal. Além disso, a presença de DM e a TFGe foram fatores independentes associados à mortalidade.

O desfecho renal foi associado ao comprimento renal total medido por US e TFGe. Sabe-se que pacientes com DRPAD com rins maiores iniciam a diálise precocemente^12,16,17^. Uma revisão sistemática^18^ constatou que idade e volume renal total foram os indicadores mais frequentemente associados à progressão da DRPAD, seguidos pela taxa de filtração glomerular estimada ou medida. Embora a maioria desses estudos tenha utilizado a medição do volume renal, tanto os valores lineares do maior eixo renal quanto os do volume renal (ambos avaliados por US e ressonância magnética) foram associados a uma evolução mais rápida da doença renal crônica (DRC)^19^. Além disso, é importante observar que nosso estudo utilizou medições de US realizadas na rotina clínica do hospital, o que reflete que a dimensão renal simples obtida na “vida real” foi capaz de prever o prognóstico. Buthani et al.^19^, mencionados acima, destacaram que rins maiores que a média de 16,5 cm apresentam o melhor ponto de corte para predizer o desenvolvimento de DRC estágio 3, enquanto nosso estudo mostrou um ponto de corte para o desfecho renal de 30 cm do comprimento renal total, ou seja, aproximadamente 15 cm em cada rim. Cornec-Le Gall e Le Meur^20^ argumentam contra o valor do comprimento renal para predizer o prognóstico na DRPAD. Nossos dados, no entanto, apontaram favoravelmente para o comprimento renal como um marcador prognóstico válido.

O aumento no comprimento renal total pode prever a progressão para o desfecho renal mesmo antes da queda da taxa de filtração glomerular. Aparentemente, a filtração glomerular é mantida por meio da hiperfiltração dos néfrons remanescentes, e a medição da TFGe pode mascarar a verdadeira perda de função dos néfrons^21^.

Os níveis séricos de ácido úrico também foram associados ao desfecho renal. Há evidências de uma associação de níveis elevados de ácido úrico com o início precoce de hipertensão, maior volume renal e risco aumentado de DRET em pacientes com DRPAD, independentemente de sexo, índice de massa corporal e função renal^22^. Foi descrito que níveis séricos mais elevados de ácido úrico são um fator de risco para disfunção endotelial em pacientes com DRPAD, mesmo em estágios iniciais^23^. O ácido úrico pode estar associado a um aumento nos mediadores pró-inflamatórios, como fator de necrose tumoral (TNF-^α^), quimiocinas^24^ e PCR^25^, o que pode levar à fibrose do parênquima renal e à progressão da doença renal. O ácido úrico prejudica a síntese de óxido nítrico em células endoteliais cultivadas^26,27^, e está associado ao aumento da atividade pró-oxidativa que pode contribuir para a disfunção endotelial^28-30^. Na DRPAD, a disfunção endotelial pode levar ao aumento da resistência vascular renal e a uma consequente diminuição do fluxo sanguíneo renal que precede o declínio da taxa de filtração glomerular e, portanto, pode prever a progressão da doença renal mesmo em níveis normais de filtração glomerular^31^. Como a elevação do ácido úrico é comum na síndrome metabólica, e a obesidade e a síndrome metabólica estão associadas à progressão da DRPAD^32,33^, é pertinente a ideia de que a síndrome metabólica possa ser explicada, pelo menos em parte, pela associação entre ácido úrico e desfecho em nosso estudo.

Reed et al.^34^ descobriram que DM e TFGe foram independentemente associados ao óbito. Os pacientes com DRPAD e DM tipo II apresentam volumes renais mais elevados, diagnóstico mais precoce de hipertensão e podem morrer em uma idade mais jovem em comparação com pacientes com doença renal policística isolada^34^. Complicações cardiovasculares são as principais causas de óbito na DRPAD, como observado em pacientes com DM^35,36^. Embora Patch et al.^37^ não tenham visado o DM como um fator prognóstico, eles descobriram que o DM foi identificado como um marcador prognóstico, e a mortalidade foi significativamente maior em pacientes com doença renal policística que eram diabéticos^37^. Possivelmente, em pacientes com DRPAD, mesmo com função renal normal, existe um comprometimento da função das células beta pancreáticas, promovendo uma secreção anormal de insulina^38^. Além disso, esses pacientes provavelmente apresentam uma redução acentuada na sensibilidade à insulina^39^, o que pode ser devido a anormalidades na membrana e no citoesqueleto que ocorrem na doença^40^. No entanto, Pietrzak-Nowacka et al.^38^ não encontraram resistência à insulina em seu trabalho. Portanto, essa última afirmação ainda não é consenso na literatura^38^.

É necessário reconhecer algumas limitações do presente estudo, como o pequeno tamanho da amostra, embora a amostra analisada tenha sido suficiente para identificar fatores medidos na rotina clínica como preditores dos desfechos em pacientes com DRPAD^41^. A ressonância magnética não estava disponível no momento do diagnóstico de nossos pacientes para uma medição mais precisa do comprimento renal total; no entanto, identificamos que a medição por US tem um valor prognóstico, a qual é de fácil acesso nos serviços de saúde. O cálculo do volume renal pela equação do elipsoide não foi utilizado neste estudo, pois não dispúnhamos de dados completos sobre a espessura e largura renal, uma vez que os exames utilizados neste estudo não foram feitos especificamente para este trabalho. No entanto, nosso estudo identifica que a medição do comprimento renal total em exames clínicos de rotina é capaz de predizer o prognóstico de pacientes. Além disso, não temos um diagnóstico genético de DRPAD para avaliar o valor prognóstico de diferentes mutações. Entretanto, essa análise é incomum na prática clínica, uma vez que poucas instalações em países em desenvolvimento têm acesso a esse recurso. Por fim, não tínhamos certeza sobre o histórico familiar de todos os pacientes. Porém, quando não dispúnhamos de informações familiares sobre um paciente, incluíamos somente aqueles com mais de 20 cistos e comprimento renal superior a 13 cm, de acordo com Iliuta et al^14^.

Como ponto forte, pudemos identificar que os dados clínicos e laboratoriais de pacientes com DRPAD de uma coorte brasileira foram associados à progressão da doença renal. Encontramos uma associação independente do comprimento renal total, taxa de filtração glomerular e níveis séricos de ácido úrico com a progressão para desfechos renais. Além disso, houve uma associação independente entre a presença de diabetes mellitus e a taxa de filtração glomerular com a mortalidade.

Em conclusão, este estudo longitudinal identificou associações entre variáveis clínicas e laboratoriais com desfechos renais e mortalidade em pacientes com DRPAD. Esses marcadores podem facilmente ajudar a prever a progressão dessa doença, indicando a necessidade de um acompanhamento mais precoce e mais próximo. Além disso, esses achados corroboram a hipótese de que tais fatores também são importantes preditores de prognóstico em uma coorte brasileira.
